# Patient satisfaction following orthodontic treatment and its association with ABO Model Grading System: a cross-sectional study

**DOI:** 10.1186/s12903-026-07980-w

**Published:** 2026-03-23

**Authors:** Yomna Abdallah Elfaisal, Amira Ahmed Aboalnaga, Mona M. Salah Fayed

**Affiliations:** 1 Orthodontic Department, Faculty of Dentistry, Cairo University, Cairo, Egypt; 2 Department of Orthodontics, Faculty of Dentistry, Cairo University, Al Saraya Str. 11, Manial, Cairo, Egypt

**Keywords:** Likert scale, Malocclusion, Objective measures, Orthodontics, Patient Satisfaction, Questionnaire

## Abstract

**Background:**

Dental appearance significantly impacts self-perception, yet clinician-based indices often fail to capture the subjective nature of patient satisfaction. Despite the importance of patient-reported outcomes, their relationship with objective measures like the American Board of Orthodontics (ABO) Model Grading System remains under investigated. This study aimed to assess post-orthodontic patient satisfaction and determine its association with objective treatment outcomes, as well as various demographic and clinical factors.

**Methods:**

This observational cross-sectional analytical study aimed to assess post-orthodontic patient satisfaction and to examine its association with objective treatment outcomes measured using the American Board of Orthodontics Model Grading System. A total of 197 patients (≥ 16 years) who completed orthodontic treatment at Cairo University were included. A cross-culturally adapted and validated questionnaire assessing ten satisfaction domains was administered using a 5-point Likert scale. The total satisfaction score was calculated as the mean of all items. Associations between satisfaction and demographic, clinical, and psychosocial factors were analyzed. Malocclusion classification and final treatment outcomes were evaluated using ABO model grading to correlate subjective satisfaction with objective measures.

**Results:**

Class I malocclusion was the most common (50.7%), followed by Class II (34.5%) and Class III (14.7%). Overall, 81.7% of participants reported being satisfied or very satisfied, with a mean satisfaction score of 4.24 out of 5. Younger patients (< 20 years) demonstrated significantly higher satisfaction levels (*p* < 0.01). No significant associations were observed between satisfaction and gender, type of malocclusion, or ABO outcome grading. Motivation, pre-treatment concerns, and treatment-related discomfort were not significantly correlated with overall satisfaction.

**Conclusions:**

Both adolescents and adults report high levels of satisfaction following orthodontic treatment, with adolescents reporting the greatest satisfaction. Patient-perceived success is not solely determined by objective clinical outcomes, highlighting the importance of addressing patient expectations and experiences during treatment.

**Trial registration:**

This study was registered at clinicaltrials.gov on [15-06-2021] with the registration number NCT04928768.

## Introduction

Dental appearance plays a crucial role in facial attractiveness and social interactions, making malocclusion a condition with important functional, psychosocial, and public health implications [1]. Malocclusion is among the most prevalent oral health problems worldwide and is associated with discomfort, reduced quality of life, and an increased risk of dental and periodontal disease [2]. Reported prevalence ranges from 40% to 93%, varying according to ethnicity, age group, and assessment methods. Although orthodontic treatment aims to restore function and esthetics, it may involve discomfort and does not always meet patient expectations [3]. With the growing number of adolescents and adults seeking orthodontic treatment, evaluation of treatment success has expanded beyond purely morphological outcomes.

Traditionally, orthodontic treatment outcomes have been assessed using clinician-based indices such as the Dental Aesthetic Index (DAI) [4], the Index of Orthodontic Treatment Need (IOTN) [5], and the American Board of Orthodontics (ABO) model grading system [6]. While these indices provide objective measures of treatment quality, they do not adequately capture patient perspectives. Since healthcare services primarily aim to benefit patients, treatment success should also be evaluated through subjective outcomes, including patient satisfaction and oral health–related quality of life (OHRQoL) [7]. Patient satisfaction is influenced by several factors, such as age, gender, treatment duration, compliance, and psychosocial characteristics, and is considered a key indicator of perceived treatment success [8–10]. Nevertheless, systematic reviews have highlighted limited and inconsistent evidence regarding the determinants of long-term satisfaction following orthodontic treatment [11, 12].

Questionnaires are widely used to assess patient-reported outcomes; however, unlike randomized clinical trials, standardized reporting frameworks for questionnaire-based studies are lacking [13]. This may result in methodological limitations and reduced data quality. The development or translation of questionnaires therefore requires careful evaluation of psychometric properties and cultural appropriateness, as validated instruments may not always be available in the target language or population [14]. In cross-cultural research, rigorous adaptation procedures are essential to preserve conceptual equivalence and minimize bias, even when instruments are translated between closely related languages [15, 16].

In recent years, cross-cultural validation of oral health–related questionnaires has gained increasing importance. Studies conducted in Europe [17–19], Asia [20], and Latin America [21] have demonstrated that cultural factors influence patients’ perceptions of esthetics, discomfort, and satisfaction with orthodontic treatment. These findings underscore the importance of using culturally adapted instruments rather than directly adopting tools developed in different settings, as cultural norms and expectations can significantly affect patient-reported outcomes [22].

Standardized adaptation procedures, including forward and backward translation, expert committee review, pretesting, and psychometric validation, are strongly recommended [23]. Validation should assess multiple aspects of measurement quality, such as content, construct, criterion, and face validity, as well as reliability parameters including internal consistency and test–retest reliability [24, 25]. These steps ensure questionnaires consistently capture patient perspectives, enabling meaningful evaluation of orthodontic outcomes across populations.

The specific objective of this study was to assess the overall level of post-orthodontic treatment satisfaction using a cross-culturally adapted and validated instrument. Furthermore, the study aimed to analyze the association between patient-reported satisfaction and objective treatment outcome measured via the ABO Model Grading System as well as various demographic and clinical determinants.

## Methods

### Study design and trial registration

The study is an observational cross-sectional analytical study that assessed the overall level of treatment satisfaction among orthodontic patients and its correlation with host factors, treatment motivation, concerns, and discomfort during/after treatment. It was registered at clinicaltrials.gov with the registration number NCT04928768 on the 15th of June, 2021. The study protocol was approved by the ethics committee of the Faculty of Dentistry, Cairo University with ID 10,221. The STROBE guidelines for clinical trials were followed. Every participant completed a written informed consent form after being fully informed of the study’s goals and specifics. The primary outcome of the current study was the overall level of orthodontic treatment satisfaction. While the secondary outcome was correlation between patient’s treatment satisfaction (Qualitative measures) and ABO grading of the finished cases (Quantitative measures).

### Sample size calculations

The study size was determined by a precision-based sample size calculation (for the primary descriptive outcome) to estimate the prevalence of total patient satisfaction. Based on the prevalence reported by Lee et al. [26] in which total patient satisfaction was (84.9%), by adopting a 95% confidence interval and a 5% margin of error with finite population correction, the predicted sample size (n) required was found to be 197 cases. Sample size calculation was performed using Epi info for windows version 7.2 (Dean et al. 2011).

### Patient selection and data collection

Collection of post-orthodontic patients’ data was conducted through the orthodontic department patient’s database. Subjects were selected following the inclusion criteria:

- Male and female patients with different malocclusions who received and completed their fixed orthodontic treatment.
- Age: 16 years old or above.
- Patients who have completed their orthodontic treatment between November 2020 and November 2021.

The primary investigator (Y.F.) contacted eligible subjects by telephone for follow-ups after orthodontic treatment and conducted the survey through personal interviews (interviewer-based questionnaire). Although it has been suggested that the specific format of survey administration does not significantly influence the stability of repeated patient-reported outcome measurements [27]. This interviewer-based approach was chosen over self-administration to ensure that all items were fully understood by the participants and to guarantee the completeness of the 10-item satisfaction scale. Furthermore, this method allowed the investigator to assist participants who might have had difficulty with specific terminology, thereby reducing the risk of missing data or incorrect interpretations.

**Fig. 1:**
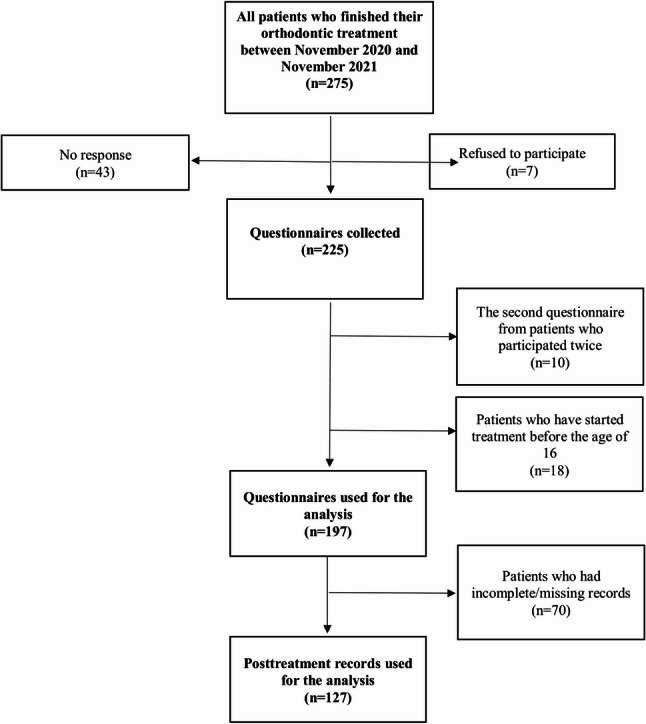
Flow diagram of the questionnaire collection and selection process

Out of the 275 patients called, 43 patients have not responded and 7 patients refused to participate in the study (total of 225). 10 subjects had 2 visits during the questionnaire period and participated twice. These data were used to calculate interrater reliability, but the results from the first survey were only included for the final calculation. After the survey period, the primary examiner retrospectively investigated the age when orthodontic treatment began and the total treatment duration for each subject. Subjects who started orthodontic treatment before the age of 16 years were excluded (*n* = 18). The questionnaires of subjects who initiated orthodontic treatment at age 16 years or older were included for the analysis (Fig. 1).

### Treatment protocol

A single, comprehensive questionnaire that was adapted from Lee et al. [26] was utilized to evaluate the patient sample. The instrument was structured into four specialized sections to capture different dimensions of the patient experience.

- Section 1: Assessed psychological motivation for seeking orthodontic treatment.
- Section 2: Evaluated expected concerns the patient held prior to beginning treatment.
- Section 3: Documented levels of discomfort experienced both during and after the treatment process.

Sections 1 through 3 utilized a multichotomous closed-ended format where patients ranked their responses, taking into consideration that there can be multiple responses. An “other” item was included to allow unexpected responses. For the sake of statistical analysis, only the first response was used. Section  4: Primarily measured the level of satisfaction after orthodontic treatment using 10 items scored on a 5-point Likert Scale. The items evaluated overall satisfaction, satisfaction with tooth alignment, facial appearance, eating and chewing, confident smile and self-image, the level of satisfaction with the retention state, treatment duration, costs, intention to recommend orthodontic treatment to others and relief of previous concerns related to orthodontic treatment.

**Fig. 2:**
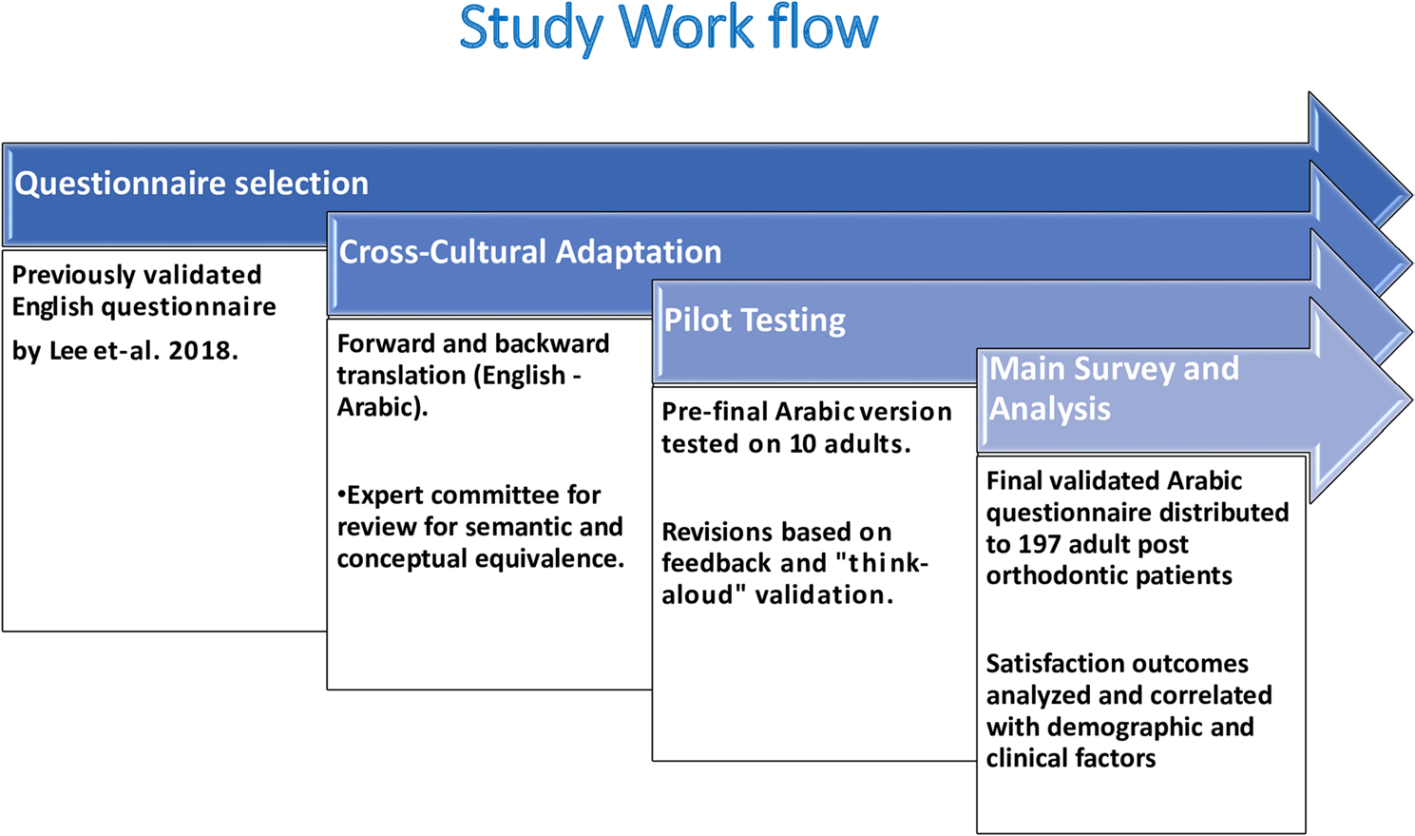
Graphical summary of the study workflow showing the development, cross-cultural adaptation, validation, and application of the orthodontic satisfaction questionnaire

The English version of the questionnaires was translated and cross-culturally adapted into Arabic following internationally recognized guidelines [25]. Forward translation was performed independently by two bilingual translators, followed by blind back-translation by two additional translators to ensure linguistic and conceptual equivalence. An expert committee consisting of methodologists, clinicians, and translators reviewed all versions and resolved discrepancies to produce a pre-final Arabic version. The pre-final questionnaire was pilot tested on ten adult patients using a “think-aloud” protocol to confirm clarity, cultural relevance, and face validity. Minor wording modifications were made based on participant feedback. Psychometric testing was conducted with a pilot group of 20 adults to assess the internal consistency and test–retest reliability of the Arabic version of the questionnaire. Internal consistency was evaluated using Cronbach’s alpha, while test–retest reliability for ordinal and nominal items was assessed using Kendall’s tau, Spearman’s rho, and Kappa statistics. Ten subjects participated in the survey twice to facilitate these reliability calculations. The final validated Arabic version was then administered to 197 adult post-orthodontic patients under the supervision of the primary investigator, following informed consent procedures (Fig. 2).

**Fig. 3:**
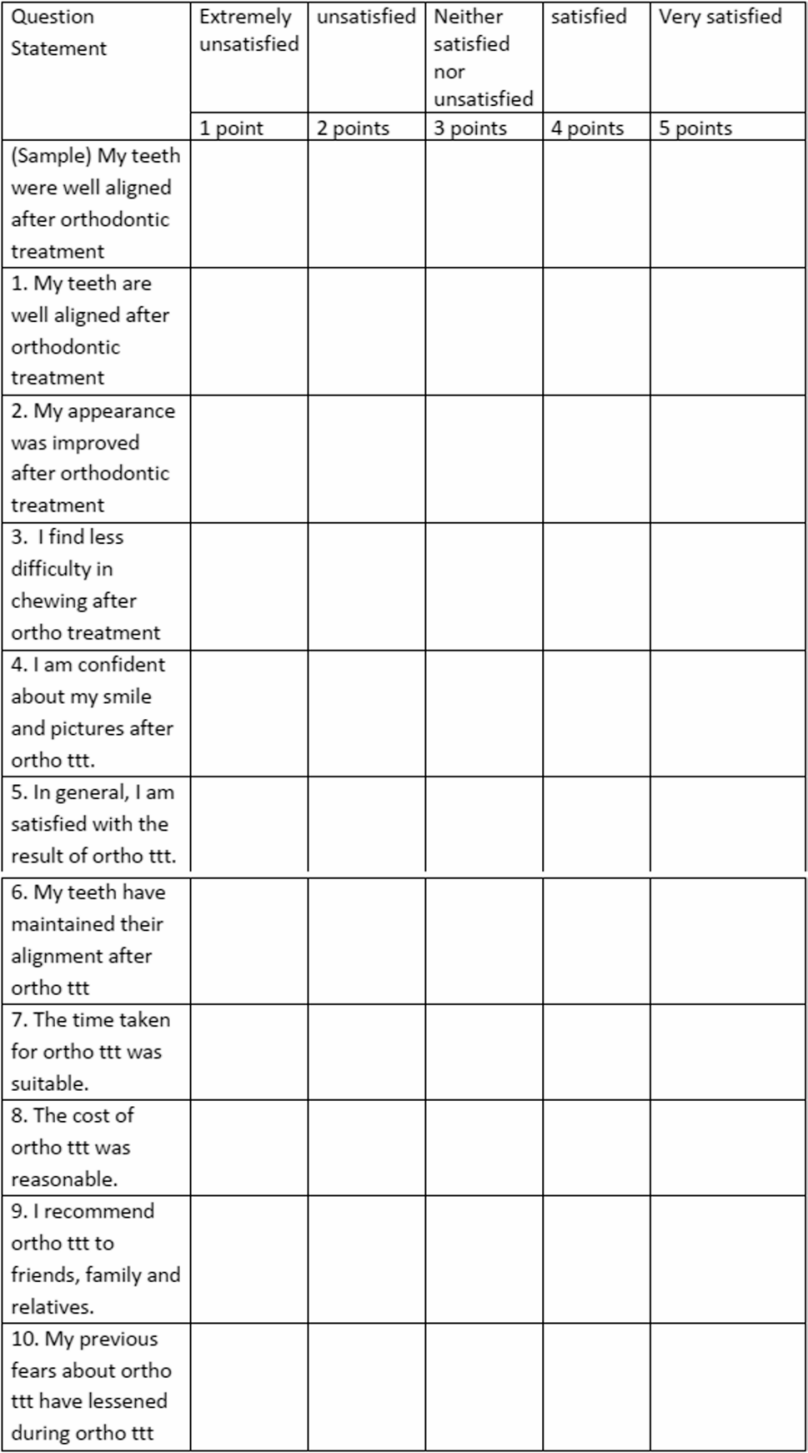
Questionnaire to assess treatment satisfaction of post-orthodontic patients

Patients were asked to mark extremely unsatisfied to very satisfied as illustrated in the first sample (Fig. 3). If the patient believes that he/she is extremely satisfied or satisfied with the alignment of his teeth, a mark will be given in the 5 or 4 points columns accordingly. If they do not agree or strongly disagree with the statement, he/she will place a mark in the 2- or 1-point column. If the answer is neither satisfied nor unsatisfied, 3 points will be chosen for that statement. Total satisfaction is calculated by averaging the Likert scores for each of the 10 items, very dissatisfied (1 point) to very satisfied (5 points). Satisfaction ratio is defined as the summated ratio of very satisfied and satisfied for each item of the questionnaire divided by the total value for all categories of the same item as follows:

$$\:\frac{\mathrm{T}\mathrm{o}\mathrm{t}\mathrm{a}\mathrm{l}\:\mathrm{v}\mathrm{a}\mathrm{l}\mathrm{u}\mathrm{e}\:\mathrm{o}\mathrm{f}\:\mathrm{v}\mathrm{e}\mathrm{r}\mathrm{y}\:\mathrm{s}\mathrm{a}\mathrm{t}\mathrm{i}\mathrm{s}\mathrm{f}\mathrm{i}\mathrm{e}\mathrm{d}+\mathrm{s}\mathrm{a}\mathrm{t}\mathrm{i}\mathrm{s}\mathrm{f}\mathrm{i}\mathrm{e}\mathrm{d}}{\mathrm{T}\mathrm{o}\mathrm{t}\mathrm{a}\mathrm{l}\:\mathrm{v}\mathrm{a}\mathrm{l}\mathrm{u}\mathrm{e}\:\mathrm{f}\mathrm{o}\mathrm{r}\:\mathrm{a}\mathrm{l}\mathrm{l}\:\mathrm{c}\mathrm{a}\mathrm{t}\mathrm{e}\mathrm{g}\mathrm{o}\mathrm{r}\mathrm{i}\mathrm{e}\mathrm{s}\:(\mathrm{V}\mathrm{e}\mathrm{r}\mathrm{y}\:\mathrm{S}\mathrm{a}\mathrm{t}\mathrm{i}\mathrm{s}\mathrm{f}\mathrm{i}\mathrm{e}\mathrm{d}-\:\mathrm{S}\mathrm{a}\mathrm{t}\mathrm{i}\mathrm{s}\mathrm{f}\mathrm{i}\mathrm{e}\mathrm{d}-\:\mathrm{N}\mathrm{e}\mathrm{u}\mathrm{t}\mathrm{r}\mathrm{a}\mathrm{l}-\:\mathrm{U}\mathrm{n}\mathrm{s}\mathrm{a}\mathrm{t}\mathrm{i}\mathrm{s}\mathrm{f}\mathrm{i}\mathrm{e}\mathrm{d}-\:\mathrm{E}\mathrm{x}\mathrm{t}\mathrm{r}\mathrm{e}\mathrm{m}\mathrm{e}\mathrm{l}\mathrm{y}\:\mathrm{u}\mathrm{n}\mathrm{s}\mathrm{a}\mathrm{t}\mathrm{i}\mathrm{s}\mathrm{f}\mathrm{i}\mathrm{e}\mathrm{d})}$$

Basic demographic information was collected at the end of the survey (gender, age, first language, level of education, date of start of orthodontic treatment, and source of referral). No other personal data were collected.

### Objective treatment outcome assessment

**Fig. 4:**
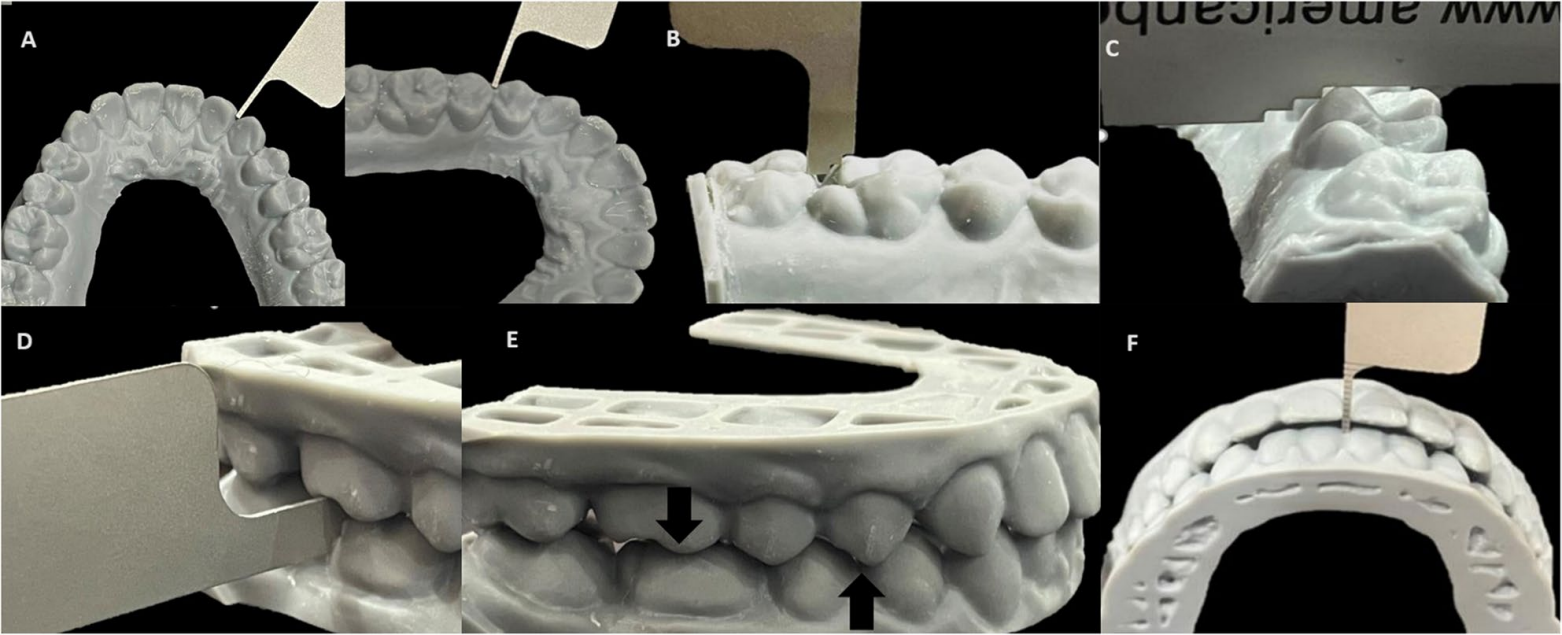
Measurements using ABO measuring gauge. **a** Alignment measurements **b**) Marginal ridges measurements. **c**) Buccolingual inclination measurements **d**) Alignment measurements. **e)** Occlusal relationship measurement **f**) Overjet measurement using ABO measuring gauge

Post-treatment records from 127 patients along with their panoramic radiographs were included. Selection was done using only 3 criteria: 1- No deciduous teeth were present, 2- No edentulous spaces were present, and 3- The models of each patient had acceptable molar and canine relationships, overjet, and overbite on visual inspection. The sample included digital STLs that were printed into resin models to be scored using the ABO measuring gauge. All the cases were evaluated by the same examiner using the American Board of Orthodontics Model Grading System (ABO-MGS) in all eight categories [28] (Fig. 4).

1. *Alignment/Rotations*: Evaluation of the incisal edges and occlusal surfaces of the teeth to ensure a smooth arch form.
2. *Marginal Ridges*: Assessment of the vertical level of the marginal ridges of posterior teeth (premolars and molars).
3. Buccolingual Inclination: Measurement of the transverse tilt of the posterior teeth using a gauge to ensure proper crown inclination.
4. *Overjet*: Assessment of the horizontal relationship of the maxillary and mandibular arches.
5. *Occlusal Contacts*: Evaluation of the contact between the functional cusps of the maxillary and mandibular posterior teeth.
6. *Occlusal Relationship*: Assessment of the anteroposterior molar and canine relationships (e.g., Class I).
7. *Interproximal Contacts*: Examination of the tightness of the contacts between adjacent teeth.
8. *Root Angulation*: Evaluation of the parallelism of the roots using the post-treatment panoramic radiograph.

Points are deducted for any deviations from the ideal; thus, a lower total score indicates a higher quality of treatment. In this study, a score of 27 or less was classified as a high-quality (low score) outcome, while scores above 27 were considered compromised (high score).

Efforts were made to minimize potential bias. Information bias was mitigated through two primary methods:

1. The use of a cross-culturally adapted and psychometrically validated questionnaire ensuring conceptual equivalence and consistency of subjective measurement.
2. The objective outcome assessment using ABO-MGS was performed by a single, calibrated examiner. Although the examiner (Y.F.) performed the interviews, the ABO-MGS was scored from models retrospectively and objectively, and the examiner was blinded to the patient’s satisfaction score during the ABO scoring process to prevent outcome expectation bias.

Recall bias was minimized as well by ensuring all participants were surveyed at least three months after debonding, encompassing different stages of the retention phase.

Selection bias due to non-response was documented; of the 275 eligible patients called, 197 participated (71.6% response rate).

### Statistical analysis

All data were collected, tabulated, and subjected to statistical analysis. Statistical analysis was performed by SPSS in general (version 20), while Microsoft Office Excel is used for data handling and graphical presentation. Qualitative categorical variables are described by frequencies and percentages. Quantitative variables are described by the Mean and Standard Deviation (SD) Shapiro-Wilk test of normality is used to test the normality hypothesis of all quantitative variables for further choice of appropriate parametric and nonparametric tests.

The total satisfaction scale is found to be not normally distributed. Hence, Kruskal-Wallis Non-Parametric test is used for comparing several groups, while for comparing two groups Mann-Whitney Non-Parametric test is applied. For correlation analysis, Spearman’s rho Correlation Coefficient is applied. Mostly the figures are presented in the Pareto format sorted from largest to smallest item to facilitate description and interpretation. The significance level is considered at *P* < 0.05 (S); while *P* < 0.01 is considered highly significant (HS). Two-Tailed tests are assumed throughout the analysis for all statistical tests.

## Results

The study included a pilot validation phase with 20 adult patients and a main cohort of 197 patients (132 females, 65 males; mean age 21.2 ± 5.1 years, range 16–46).

### Test-Retest Reliability (Nominal Data)

For nominal items, percentage agreement and Kappa coefficients were calculated. Kappa values indicated substantial to almost perfect agreement, ranging from 0.607 (“What bothered you the most”) to 0.882 (“What made you the most uncomfortable”), with percentage agreement between 72.2% and 95% (Table 1). Specifically, the item assessing the purpose/motive for treatment showed substantial reliability (Kappa = 0.740, *P* < 0.001; 80% agreement).

**Table 1:** Test-Retest for nominal data

Questionnaire Item	Kappa Value	*P* Value	Percentage of Agreement
**Purpose/motive for considering orthodontic treatment**	0.740	0.000	80%
**What bothered you the most**	0.607	0.000	72%
**What made you the most uncomfortable**	0.882	0.000	95%

### Internal consistency

Cronbach’s alpha for the 10-item Likert scale measuring satisfaction was 0.698, indicating acceptable internal consistency.

### Test-Retest Reliability (Ordinal Data)

For the 10-item Likert scale, Kendall’s tau and Spearman’s rho were calculated. Most items showed strong reliability (ρ > 0.60), while “appearance” and “smile confidence” demonstrated moderate reliability (ρ = 0.50) (Table 2).

**Table 2:** Test-Retest reliability of the ordinal data

Questionnaire Item	Kendall’s tau		Spearman’s rho	
	Value	*P* Value	Value	*P* Value
1. **My teeth are well aligned**	0.771	0.00028	0.777	0.00006
2. **My appearance has improved**	0.503	0.02849	0.503	0.02394
3. **I have less trouble chewing**	0.835	0.00003	0.866	0.00000
4. **I have confidence at my smile**	0.487	0.02436	0.509	0.02183
5. **Overall**,** I am satisfied with**	0.748	0.00033	0.792	0.00003
6. **My teeth have maintained their well-aligned**	0.755	0.00016	0.794	0.00003
7. **The time taken for orthodontic**	0.946	0.00000	0.953	0.00000
8. **The charges for orthodontic**	0.832	0.00004	0.857	0.00000
9. **I would recommend orthodontic treatment**	0.582	0.00738	0.601	0.00503
10. **My previous concerns of orthodontic treatment**	0.740	0.00019	0.800	0.00002

### Main study

Among the 197 respondents, Class I malocclusion was the most common (50.8%), followed by Class II (34.5%) and Class III (14.7%). The leading motivation for treatment was to smile with confidence and make a good impression (40.6%), followed by tooth alignment (31.5%). The most frequent pre-treatment concern was pain during treatment (35.0%). Discomfort during treatment was primarily related to appliance irritation and chewing difficulty (53.8%).

### Treatment satisfaction

Overall, 61.4% of participants reported being very satisfied and 20.3% satisfied, giving a total satisfaction ratio of 81.7% and a mean satisfaction score of 4.24 out of 5. The highest satisfaction was reported for confidence in smile (89.9%) and recommendation of treatment to others (89.3%), while the lowest satisfaction was related to treatment duration (61.9%) and retention stability (67.0%) (Table 3).

**Table 3:** Satisfaction subscales and total satisfaction scale

	Very dissatisfied		Dissatisfied		Neutral		Satisfied		Very satisfied	
	f	%	f	%	f	%	f	%	f	%
My teeth are well aligned	4	2.03	6	3.05	20	10.15	77	39.09	90	45.69
My appearance has improved after treatment	4	2.03	0	0.00	20	10.15	60	30.46	113	57.36
I have less trouble chewing after treatment.	14	7.11	2	1.0	50	25.38	44	22.34	87	44.16
I have confidence at my smile and image after treatment	3	1.5	2	1.02	15	7.61	56	28.43	121	61.4
Overall, I am satisfied with the result of treatment.	3	1.52	8	4.0	25	12.69	40	20.30	121	61.4
My teeth have maintained their well-aligned state since the end of treatment.	6	3.05	16	8.12	43	21.83	65	32.99	67	34.0
The time taken for treatment was appropriate.	7	3.55	14	7.1	54	27.41	53	26.90	69	35.03
The charges for treatment are moderate.	0	0.00	5	2.54	21	10.66	47	23.86	124	62.94
I would recommend orthodontic treatment to friends, family or relatives.	2	1.02	2	1.0	17	8.63	25	12.69	151	76.65
My previous concerns were relieved after treatment.	4	2.03	4	2.03	48	24.37	40	20.30	101	51.27
Total satisfaction scale	47	2.39	59	2.99	313	15.89	507	25.74	1044	52.99

### ABO grading

Results were categorized into two ABO grading groups: ≤27 (low score) and > 27 (high score). Participants with low ABO scores (≤ 27) had slightly higher mean satisfaction (4.30 ± 0.46) than those with high ABO scores (> 27; 4.15 ± 0.58). The Mann-Whitney U test indicated that this difference was not statistically significant (U = 1853.5, Z = − 1.083, *P* = 0.278). Spearman’s correlation confirmed an extremely weak, non-significant relationship between ABO grading and total satisfaction (rs = 0.01, *P* = 0.91), indicating minimal impact of ABO scores on patient-reported satisfaction (Table 4).

**Table 4:** Relation between ABO grading and total satisfaction

ABO Grading Group	*N*	Mean Satisfaction ± SD	Mann-Whitney U	Z	*P* Value	
≤ 27 (low score)	95	4.30	1853.500	-1.083	0.27881	*P* > 0.05 NS
> 27 (high score)	32	4.15				

### Age and Satisfaction

The relationship between age and total satisfaction was analyzed. Age showed a weak but statistically significant negative correlation with overall satisfaction (Spearman’s rho = − 0.21, *p* < 0.01), indicating higher satisfaction among younger patients. Mean satisfaction scores for each age group are shown (Table 5).

**Table 5:** Relation between age and total satisfaction

Age	*N*	Mean	SD	Spearman’s rho (rs​)	*p*-value
< 20	103	4.32	0.44		
20 to 29 years	80	4.16	0.52		
≥ 30	14	4.11	0.63		
Overall	197	4.24	0.51	-0.21	0.00258

Independent t-test analysis comparing adolescents (< 20 years) with adult patients (≥ 20 years) confirmed that adolescents had significantly higher mean satisfaction scores (4.30 ± 0.45 vs. 4.16 ± 0.50, *P* = 0.00258). These results indicate that younger patients reported slightly higher satisfaction with orthodontic treatment, although the effect was relatively weak.

### Associations between satisfaction and patient characteristics

The relationships between total patient satisfaction and demographic and treatment-related variables were examined. No significant differences were observed for gender (males: 4.25 ± 0.44; females: 4.24 ± 0.52; Mann-Whitney U = 4137.5, *P* = 0.883; point-biserial *r* = 0.08, *P* = 0.25), malocclusion type (Class I: 4.16 ± 0.54; Class II: 4.34 ± 0.38; Class III: 4.30 ± 0.50; Kruskal-Wallis *P* = 0.137; Spearman’s rho = 0.05, *P* = 0.45), treatment motivation (range 4.15–4.29; Kruskal-Wallis *P* = 0.975; Spearman’s rho = 0.12, *P* = 0.09), pre-treatment concerns (range 3.99–4.44; Kruskal-Wallis *P* = 0.338; Spearman’s rho = − 0.10, *P* = 0.12), or treatment-related discomfort (range 4.14–4.29; Kruskal-Wallis *P* = 0.614; Spearman’s rho = − 0.14, *P* = 0.06). These results indicate that, aside from age, none of the examined variables were significantly associated with overall satisfaction.

## Discussion

Orthodontic treatment aims not only to improve dental appearance and function but also to enhance psychosocial wellbeing. Patient satisfaction, defined as “the positive evaluation of distinct dimensions of healthcare” [29] has therefore become an essential outcome measure in orthodontics, reflecting the perceived quality of care and treatment success [30]. Untreated malocclusion negatively affects psychological, social, and physical wellbeing, reducing oral health-related quality of life [31].

Recent research has emphasized the importance of incorporating patient-reported outcomes rather than relying solely on clinician-based measures [32]. Factors such as age, gender, psychological traits, compliance, treatment duration, and dentofacial improvement can influence satisfaction levels [33, 34] However, evidence regarding these determinants remains inconsistent. factors, motivation, concerns, and discomfort during or after treatment.

The overall satisfaction rate in this study (81.7%) aligns with previous reports showing satisfaction rates between 74% and 96% [10, 26, 35]. Most participants were highly satisfied with their smile and appearance but less so with treatment duration and retention stability. These findings support previous studies reporting that challenges experienced during treatment are outweighed by the perceived esthetic and functional improvements [10, 36].

A significant negative correlation was found between age and satisfaction, indicating that younger patients were generally more satisfied with treatment outcomes. Similar findings were reported by Al-Omiri & Abu Alhaija [37] and contradict those of Lee et al. [26], who observed higher satisfaction in adults. No significant gender differences were detected, consistent with Maia et al. [12] and Feldmann [38], suggesting that satisfaction is independent of sex.

The main motivation for treatment was to improve smile esthetics and self-confidence, consistent with previous reports emphasizing the dominance of aesthetic motives among orthodontic patients [39, 40]. Most patients-initiated treatment by personal choice, highlighting intrinsic motivation as a positive predictor of treatment satisfaction [41, 42]. Pre-treatment concerns were primarily related to pain and treatment duration, findings that agree with Bradley et al. [10]. While a study by the most common pre-treatment concerns were alignment and being embarrassed to smile [43].

Discomfort during treatment was mostly associated with appliance irritation and difficulty in chewing, in line with Al-Omiri & Abu Alhaija [37] and Zhou et al. [44], who noted that discomfort peaks shortly after appliance insertion. However, these temporary issues did not significantly affect overall satisfaction. No significant correlations were found between satisfaction and motivation, concerns, or discomfort, similar to the findings of van Wezel et al. [45].

Several studies have emphasized that the doctor–patient relationship is one of the strongest determinants of satisfaction, outweighing clinical outcomes [34, 43, 46]. Although not directly measured in this study, patient-centered communication and professional conduct likely contributed to the high satisfaction levels observed. Furthermore, the extremely weak correlation between satisfaction and the ABO grading of treatment outcomes suggests that perceived satisfaction is not necessarily linked to objective measures of occlusal quality (Estella et al., 2017).

Overall, this study demonstrates a high level of satisfaction among adults following orthodontic treatment. Age was the only significant determinant, while gender, malocclusion type, motivation, concerns, and discomfort showed no significant effect. These findings highlight the complex nature of satisfaction, influenced more by individual perception and interpersonal factors than by objective treatment outcomes.

The high overall satisfaction observed in this study is consistent with previous reports. Lee et al. [26] reported an 84.9% satisfaction rate, with nearly half of patients being “very satisfied,” while Saleh et al. [37] found that 90.5% were satisfied with their post-treatment alignment. Similarly, Bradley et al. [10] reported a satisfaction rate of 96%, with most patients considering the challenges of treatment worthwhile. In contrast, Larsson and Bergström [47] reported a slightly lower rate of 74%, highlighting variability across populations. This range, which spans from approximately 34% to 95% in the literature, may be explained by differences in methodology, instruments used, and cultural or demographic factors.

An important finding of the present study was the weak association between ABO grading and patient satisfaction. This suggests that patient perceptions of treatment success are not necessarily aligned with objective clinical standards. Similar observations were reported by Estrella et al. and Maia et al. [12], who found no significant correlation between satisfaction scores and ABO or Peer Assessment Rating index outcomes (PAR). Feldmann [48] reported a weak association between satisfaction and occlusal changes, though not with final PAR scores. Collectively, these findings indicate that while orthodontic treatment improves occlusal indices, subjective satisfaction is more closely related to esthetics, psychosocial benefits, and patient expectations than to objective measures.

In this study, the ABO-MGS was utilized to provide a high-precision objective assessment of treatment outcomes [28]. While other indices, such as PAR index or the Index of Complexity, Outcome, and Need (ICON), are commonly used to measure general improvement or treatment need, they lack the sensitivity required to evaluate the fine details of a finished occlusion [49, 50]. Additionally, the system has been validated for use with both traditional plaster and digital models, supporting its applicability in contemporary orthodontic practice [51].The ABO-MGS, which is the benchmark for clinical excellence in board certification, employs a rigorous point-deduction system that identifies even minor discrepancies in tooth alignment, marginal ridges, and occlusal relationships [52].

The study design strengthened the reliability of responses, as participants were surveyed at least three months after debonding, encompassing different stages of the retention phase while minimizing recall bias. However, some patients gave neutral responses, consistent with earlier studies, which may reflect limited doctor–patient communication or insufficiently clear expectations at the start of treatment [53, 54]. This highlights the importance of shared decision-making and comprehensive informed consent to improve satisfaction levels.

The present results also contribute to the ongoing debate about determinants of satisfaction. While some studies have suggested gender or treatment duration as influential factors, no such associations were observed here. Instead, age emerged as the key determinant, possibly reflecting differences in expectations between adolescents and adults.

From a broader perspective, the consistently high satisfaction rates reported across multiple populations underscore the overall effectiveness of orthodontic treatment in improving patient-perceived outcomes. However, systematic reviews emphasize that evidence regarding long-term stability and its impact on satisfaction remains limited.

While the methodology was robust, the study does have some limitations. First, generalization of the results to different populations should be performed with caution, since the sample studied belonged to a public dental clinic at a Faculty of Dentistry, and there were inclusion and exclusion criteria that made the study sample different from the general population. Another limitation was that in the present sample, the type of orthodontic treatment has not been taken into account. The type of orthodontic treatment may affect patient satisfaction. While ABO-MGS is a clear and objective method of measuring clinical outcome, other methods such as three-dimensional laser scanning are more precise and may be able to detect differences between appliances. However, we would argue that the goal of orthodontics as a specialty is always to produce the best aesthetic result. As a result, a difference that is not visible to the naked eye is of little consequence. This underlines the need for further prospective, long-term studies to clarify the relationship between occlusal stability and patient-reported outcomes.

Reporting patients’ satisfaction offers several practical benefits beyond simply evaluating treatment outcomes. It encompasses patients’ assessments of care, expectations, and preferences, and thus provides insight into the quality of care processes as well as objective results. Reporting satisfaction enables orthodontists to better understand patient expectations, improve communication, and tailor treatment approaches to enhance the overall patient experience. Effective clinician–patient communication and trust are central to satisfaction and adherence, with quality of interaction often correlating more strongly with satisfaction than technical outcomes alone [55, 56]. Systematic assessment of satisfaction can identify areas for improvement in service delivery and practice management, which supports quality enhancement and fosters long-term patient loyalty and willingness to revisit [57]. For patients, feedback ensures that concerns are acknowledged and addressed, improving engagement, adherence, and overall satisfaction with the treatment process [58]. Incorporating patient-reported measures alongside objective clinical indices supports a more comprehensive and patient-centred evaluation of orthodontic treatment outcomes, aligning clinical practice with modern dental care priorities [59].

## Conclusion

This study found that adolescents and adults report high satisfaction following orthodontic treatment, with age being the main determinant. Satisfaction showed only a weak relationship with ABO grading, underscoring the importance of integrating patient perspectives alongside clinical outcomes.

These findings highlight the value of patient-centered approaches and culturally validated tools for evaluating orthodontic success.

## Data Availability

The datasets used in the present study are available from the corresponding author upon reasonable request.

## Abbreviations

ABO: American Board of Orthodontics
MGS: Model Grading System
PAR: Peer Assessment Rating
ODI: Orthodontic Discomfort Index
QoL: Quality of Life
